# Evaluating Smart Assistant Responses for Accuracy and Misinformation Regarding Human Papillomavirus Vaccination: Content Analysis Study

**DOI:** 10.2196/19018

**Published:** 2020-08-03

**Authors:** John Ferrand, Ryli Hockensmith, Rebecca Fagen Houghton, Eric R Walsh-Buhi

**Affiliations:** 1 School of Public Health-Bloomington Indiana University Bloomington, IN United States

**Keywords:** digital health, human papillomavirus, smart assistants, chatbots, conversational agents, misinformation, infodemiology, vaccination

## Abstract

**Background:**

Almost half (46%) of Americans have used a smart assistant of some kind (eg, Apple Siri), and 25% have used a stand-alone smart assistant (eg, Amazon Echo). This positions smart assistants as potentially useful modalities for retrieving health-related information; however, the accuracy of smart assistant responses lacks rigorous evaluation.

**Objective:**

This study aimed to evaluate the levels of accuracy, misinformation, and sentiment in smart assistant responses to human papillomavirus (HPV) vaccination–related questions.

**Methods:**

We systematically examined responses to questions about the HPV vaccine from the following four most popular smart assistants: Apple Siri, Google Assistant, Amazon Alexa, and Microsoft Cortana. One team member posed 10 questions to each smart assistant and recorded all queries and responses. Two raters independently coded all responses (κ=0.85). We then assessed differences among the smart assistants in terms of response accuracy, presence of misinformation, and sentiment regarding the HPV vaccine.

**Results:**

A total of 103 responses were obtained from the 10 questions posed across the smart assistants. Google Assistant data were excluded owing to nonresponse. Over half (n=63, 61%) of the responses of the remaining three smart assistants were accurate. We found statistically significant differences across the smart assistants (N=103, χ^2^_2_=7.807, *P*=.02), with Cortana yielding the greatest proportion of misinformation. Siri yielded the greatest proportion of accurate responses (n=26, 72%), whereas Cortana yielded the lowest proportion of accurate responses (n=33, 54%). Most response sentiments across smart assistants were positive (n=65, 64%) or neutral (n=18, 18%), but Cortana’s responses yielded the largest proportion of negative sentiment (n=7, 12%).

**Conclusions:**

Smart assistants appear to be average-quality sources for HPV vaccination information, with Alexa responding most reliably. Cortana returned the largest proportion of inaccurate responses, the most misinformation, and the greatest proportion of results with negative sentiments. More collaboration between technology companies and public health entities is necessary to improve the retrieval of accurate health information via smart assistants.

## Introduction

### Background

Voice assistants, a form of chatbot or conversational agent often referred to colloquially as “smart assistants,” are devices that respond to human voices and can be commanded to do a variety of tasks [1]. Smart assistants have existed in their most contemporary form since 2010, with the introduction of Siri, and they are used for numerous tasks such as automation (ie, integration with climate control devices and entertainment devices), retrieval of information about certain topics, and shopping [1]. Smart assistants have been integrated into smartphones, laptops, speakers, and other devices, creating a large network of consumers who frequently utilize smart assistants to acquire information on a range of topics [1].

### Prevalence of Smart Assistant Use

According to the Pew Research Center, out of a total sample of 2045 Americans, nearly half (46%) reported using smart assistants in 2017 [2]. Of 4272 Americans surveyed in 2019, one-quarter (25%) had a stand-alone smart assistant device (eg, Amazon Echo, Google Home) in their homes [3]. Households that reported having a stand-alone smart assistant device (n=1067) also reported higher income (34% earned US $75,000 or more per year; 15% earned below US $30,000) [3]. The same report indicated that younger Americans frequently use stand-alone smart assistants, with 32% of users aged between 18 and 29 years and 28% of users aged between 30 and 49 years [3]. This difference may be due to varied ways of assessing use (eg, ever use and daily use). In contrast, smartphone smart assistants are more frequently adopted by those aged between 18 and 29 years (81%), whereas those aged between 45 and 60 years report the most daily active use of smartphone smart assistants [2]. These usage trends depict nuanced usage patterns wherein younger users are the most common adopters of smartphone smart assistants and older users, once they begin using smartphone smart assistants, use them more frequently than do other age groups. These varying adoption and usage behaviors provide multiple avenues for targeting different age groups through smart assistants.

### Uses of Smart Assistants

More than half of Americans report that a major reason for using smart assistants is the ability to interact with their devices without using their hands [2]. One-quarter of Americans say that they use smart assistants to remotely control other devices such as heating systems, door locks, and lights [2]. Given that approximately 35% of Americans report searching online (ie, using Google or another search engine) to self-diagnose a medical condition, it seems logical to assume that individuals may turn to smart assistants for this information as well [4]. Unfortunately, smart assistants are relatively new technologies, and their responses have not been rigorously evaluated in many contexts.

### Previous Assessments of Smart Assistants

In general, smart assistants have been evaluated for their response accuracy [5] and their general usability [6]. While considerable research has been conducted assessing digital health approaches, such as text message–based approaches and mobile apps for a range of health behaviors [7-9], few have included smart assistant responses to health-related queries. For instance, a pilot study comparing two smart assistants (Google Assistant and Apple Siri) to a standard Google Search on the topic of smoking cessation resources found that Google Assistant provided a greater number of evidence-based responses [10]. Other studies have specifically examined consumer experiences with the natural language processing of smart assistants [5].

### Human Papillomavirus Prevention

To effectively assess smart assistant responses for accuracy and misinformation, human papillomavirus (HPV) has been identified as a controversial content area with a substantial evidence base. The evidence base of HPV is scientifically valid but hotly contested. HPV is the most common sexually transmitted infection and is a known cause of cervical cancer, as well as several other types of cancers [11]. While most sexually active people will contract HPV at some point in their lives, how the infection resolves varies from person to person, and a federally approved vaccine has been shown to be effective at reducing both the incidence of HPV transmission and the incidences of genital and anogenital HPV infection and cervical lesions [12]. Despite studies affirming the positive effects of the HPV vaccine while debunking inaccurate claims, there continues to be large-scale misinformation efforts (mostly conducted online through social media platforms) surrounding this issue, which are driven in part by the antivaccination movement [13-20]. These issues of misinformation have contributed to mistrust of medical professionals [21-23] and misunderstanding of diseases and their risks [24,25]. In January 2020, the World Health Organization (WHO) released a list of urgent global health challenges for the new decade, including stopping vaccine-preventable diseases and earning the public’s trust [26]. These two challenges are closely intertwined, as trust helps to shape whether individuals rely on provider recommendations (eg, whether parents will vaccinate their children) [22,27] and how misinformation disseminated online and across social media platforms influences vaccine refusal or vaccine delay [28]. In fact, the WHO has stated that vaccine hesitancy is one of the top 10 threats to global health [29].

In this study, we attempted to answer the following research question: do responses to smart assistant queries vary between different smart assistants, with regard to accuracy, misinformation, and sentiment toward HPV vaccination?

## Methods

### Query Development

In order to effectively assess smart assistants’ responses for accuracy and misinformation, we utilized questions from the chat-text service of Planned Parenthood (personal communication by Nicole Levitz; March 18, 2019). We chose to focus on questions around the HPV vaccine owing to the previously identified issues of accuracy and misinformation surrounding this topic [30,31]. We chose variations of 10 evidence-based questions from the Planned Parenthood system, allowing us to better evaluate responses to those questions for accuracy and misinformation. The questions are listed in Textbox 1.

**Queries posed to smart assistants.**

1. Does the HPV vaccine work?

2. Does the HPV vaccine cause autism?

3. Does Gardasil work?

4. Does Gardasil cause autism?

5. Is the HPV vaccine dangerous?

6. Is Gardasil dangerous?

7. Who can get the HPV vaccine?

8. Where can I get the HPV vaccine?

9. Does Gardasil kill?

10. How much does the HPV vaccine cost?

### Search Process

One member of the research team queried each of the four most popular smart assistants (Apple Siri, Google Assistant, Amazon Alexa, and Microsoft Cortana), which were identified in the 2019 Voice Report by Microsoft [32]. In the event that the smart assistant provided a nonresponse to a query (eg, “I don’t know how to answer that”), the team member queried the smart assistant a maximum of three times to ensure that it was not an errored response due to misunderstanding the query, background noise, or some other unrelated reason.

Smart assistants provided varying numbers of results on their respective first pages for each query. Alexa and Google Assistant provided only one oral result for each query, whereas Siri provided five text results for each query. Cortana provided between three and nine text or video results for each query. Multimedia Appendix 1 displays the number of results each smart assistant provided per query. The team member, responsible for conducting the searches, recorded the first page of the results for data extraction based on previous studies indicating that users more frequently click on the top 10 results, which tend to be concentrated on the first page of most search results [33]. There is also evidence suggesting that people do not necessarily only click the first result on a page. In a 2020 study of search engine optimization, 16% of respondents in the study reported clicking on only the first result compared with 17% and 14% of respondents who reported clicking on three and five results, respectively [34]. This finding suggests that the remaining results on the first page should be extracted to best replicate actual human search behavior.

### Recording Process

**Figure 1:**
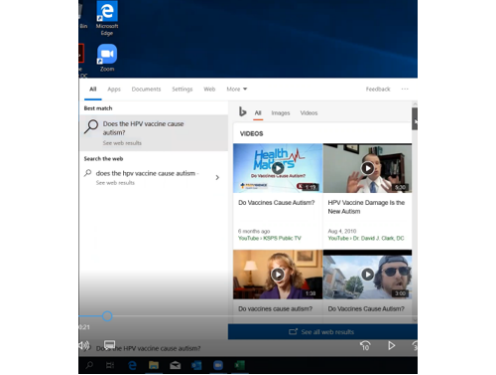
Example of a video result from a query to Cortana.

The team member, who posed questions to the smart assistants, used video and audio recording software to record both the queries and the smart assistant responses. We used these recordings in the data extraction and coding process. Figure 1 depicts a recording of a query posed to Cortana.

### Data Extraction and Coding Process

We developed a web-based (Qualtrics) survey to aid in data extraction and coding of the smart assistant responses. Survey items were designed to extract relevant data from the smart assistant responses, including the types of responses (eg, video, web page, blog, and journal article), sources of the responses (eg, business, doctors, and health information provider), sentiments of the responses toward the HPV vaccine (eg, positive, neutral, and negative), accuracy of the responses, any incidence of misinformation in the responses, and topics discussed in the responses (eg, cancer, sexual behavior, and conspiracy theory).

We defined *accuracy* as a response that reflected the existing evidence. We coded accuracy using a dichotomy approach (0 for not accurate; 1 for accurate), which we applied specifically to the query (ie, accuracy of unrelated tangential content was not considered). If the response did not answer the query with the correct reply or positioned the correct reply as dubious or incorrect, we considered it “not accurate.” We defined *misinformation* as either deliberate or accidental promotion of previously disproved or unproven beliefs, attitudes, and behaviors. We coded misinformation using a dichotomy approach (0 if it did not provide misinformation; 1 if it did provide misinformation), which we applied to the entirety of the response (not just the answer to the question posed). If any misinformation was found in the smart assistant’s response, we coded it as having provided misinformation. Table 1 depicts examples of accuracy and misinformation in smart assistant responses to several queries. Since accuracy and misinformation were coded based on different aspects of the smart assistant responses, there were some cases with both an accurate response to the query and some misinformation in the result. We coded sentiment toward vaccines as one of the following four potential categories: negative (mostly negative statements), neutral (neither positive nor negative statements), positive (mostly positive statements), and ambiguous (both negative and positive statements). We applied sentiment coding to the entirety of the response.

Two independent team members extracted and coded the data with an almost perfect agreement (κ=0.85) [35]. We resolved discrepancies through discussion.

**Table 1:** Examples of accuracy and misinformation in smart assistant responses.

Query	Accuracy	Misinformation
Does the HPV^a^ vaccine work?	“In the trials that led to the approval of Gardasil and Cervarix, these vaccines were found to provide nearly 100% protection against persistent cervical infections with HPV types 16 and 18 and the cervical cell changes that these persistent infections can cause.”	N/A^b^
Does the HPV vaccine cause autism?	“There is no link between vaccines and autism.”	“The scientific literature is now starting to fill up with case reports and studies and articles that irrefutably show that there is a connection between this vaccine (and it’s an ugly vaccine) and neurological damage.”
Does Gardasil work?	“Gardasil works by preventing the infection of the types of HPV that can lead to cervical cancer...”	“The Gardasil HPV vaccine has been proved to have caused the deaths of 32 women.”
Does Gardasil cause autism?	“There has never been a study that has shown that vaccines cause autism.”	N/A
Is the HPV vaccine dangerous?	“Findings from many vaccine safety monitoring systems and more than 160 studies have shown that HPV vaccines have a favorable safety profile—the body of scientific evidence overwhelmingly supports their safety.”	“Aluminum in the vaccines is toxic enough to be harmful.”
Is Gardasil dangerous?	“Although this information is accurate in a strictly literal sense, it is a misleading presentation of raw data that does not in itself establish a causal connection between Gardasil and the posited medical dangers.”	“The Gardasil HPV vaccine has been proved to have caused the deaths of 32 women.”
Who can get the HPV vaccine?	“All people ages 9 to 45 can get the HPV vaccine to protect against genital warts and/or different types of HPV that can cause cancer.”	N/A
Where can I get the HPV vaccine?	“The HPV vaccine is available at: Healthcare Clinic for patients aged 11-26. Walgreens Pharmacy. Ages vary by state.”	N/A
Does Gardasil kill?	“I cannot stress this enough, based on this report alone you can't make a determination that the vaccine caused the deaths.”	N/A
How much does the HPV vaccine cost?	“Each dose of the vaccine can cost about $250.”	N/A

^a^HPV: human papillomavirus.

^b^N/A: not applicable.

### Analyses

The sample consisted of 128 total data points across all four smart assistants. We excluded any nonresponse data points (eg, “I don’t know how to answer that”) and responses that were categorized as *other* (eg, responded with completely irrelevant content to the query) from the final analyses. Google Assistant provided nonresponses to every query and was removed, thus reducing our final analysis sample to 103 responses across three smart assistants. These nonresponses and other responses were removed because they did not in any way address the query posed. Multimedia Appendix 1 displays the frequencies of responses and nonresponses for each smart assistant. Descriptive frequencies and chi-square difference tests were used to examine the levels of accuracy and misinformation among the smart assistants.

## Results

Table 2 displays the source and content characteristics of the smart assistant responses. Smart assistant responses contained content published by an organization in 82 out of 103 (79.6%) responses, whereas 20 out of 103 (19.4%) responses were published by an individual. Content was provided by organizations including some type of health information provider (eg, Planned Parenthood and Mayo Clinic) in 36 out of 103 (35.0%) responses and by a government entity (eg, Centers for Disease Control and Prevention) in 25 out of 103 (24.3%) responses. Cortana provided content published by individuals (eg, physicians and journalists) in 18 out of 61 (29.5%) responses, whereas Siri and Alexa provided content published by individuals in only 2 out of 36 (5.6%) and 0 out of 6 (0.0%) responses, respectively. The most common type of individual who provided content (10 out of 103 responses, 9.7%) was some classification of physician. The type of content provided in the smart assistant responses varied, with videos provided in 30 out of 103 (29.1%) responses, driven entirely by Cortana, which was the only smart assistant to provide video responses. Siri’s responses were classified as frequently asked question (FAQ) pages in 13 out of 36 (36.1%) responses, whereas Cortana’s responses were classified as videos in 30 out of 61 (49.2%) responses and as general web pages in 12 out of 61 (19.7%) responses.

**Table 2:** Source and content characteristics of smart assistant responses.

Variable			Value (N=103), n (%)
**Source^a^**			
	Health information provider		36 (35.0)
	Federal/city/state		25 (24.3)
	Nonprofit/advocacy group		15 (14.6)
	Physician		10 (9.7)
	Other		9 (8.7)
	News/media organization		8 (7.8)
	Business		6 (5.8)
	Journalist/press		2 (1.9)
	Politician		2 (1.9)
	Health care organization		1 (1.0)
**Content**			
	**Format of the smart assistant responses**		
		Video	30 (29.1)
		General web page	24 (23.3)
		Article	14 (13.6)
		FAQ^b^ site	19 (18.4)
		Other	6 (5.8)
		Blog post	3 (2.9)
		Location/map/directions	1 (1.0)
	**Topics mentioned in the smart assistant responses^a^**		
		Cancer	77 (74.8)
		Vaccines and side effects	57 (55.3)
		Sexual behaviors	26 (25.2)
		Access to medical care	20 (19.4)
		Direct impacts on loved ones	13 (12.6)
		Conspiracy theories	6 (5.8)
		Vaccines are ineffective	6 (5.8)
		Mass media	6 (5.8)
		Risk of disease is lower than risk of adverse vaccination events	2 (1.9)
	**Sentiment expressed toward vaccines in smart assistant responses**		
		Positive	65 (63.1)
		Neutral	18 (17.5)
		Ambiguous	11 (10.7)
		Negative	7 (6.8)

^a^Totals may exceed 100% owing to the availability of multiple response options.

^b^FAQ: frequently asked question.

Tables 3 and 4 depict the differences among smart assistants in terms of primary outcomes. Smart assistant responses contained accurate answers in 63 out of 103 (61.2%) responses. Neither response accuracy (*P*=.10) nor response sentiment (*P*=.22) was significantly different among the devices. The number of responses provided for each query varied across the devices, but 4 out of 6 (66.7%) responses by Alexa and 26 out of 36 (72.2%) responses by Siri were accurate. In contrast, 33 out of 61 (54.1%) responses by Cortana were accurate. There were no cases in which responses contained both accurate answers and misinformation. Chi-square tests indicated that the three smart assistants significantly varied in terms of whether they provided misinformation in their responses (N=103, χ^2^_2_=7.807, *P*=.02) and whether they provided any evidence to support the claims made in their responses (N=103, χ^2^_2_=11.054, *P*=.004). Smart assistant responses contained at least one instance of misinformation in 18 out of 103 (17.5%) responses. Responses by Alexa contained no misinformation, whereas misinformation was present in 2 out of 36 (5.6%) responses by Siri and 16 out of 61 (26.2%) responses by Cortana. Alexa provided evidence to support each of its responses, whereas Siri provided evidence in 21 out of 36 (58.3%) responses and Cortana provided evidence in 23 out of 61 (37.7%) responses.

In general, smart assistant responses contained positive sentiment toward vaccines, with 65 out of 103 (63.1%) responses containing primarily positive sentiment. The second most common sentiment was “neutral,” which was found in 18 out of 103 (17.5%) responses. The least common sentiment was “negative,” which was found in only 7 out of 103 (6.8%) responses, and all were provided by Cortana.

**Table 3:** Response differences among smart assistants in terms of accuracy, evidence provided, and misinformation.

Response quality of the smart assistants		Value, n/N (%)	Chi-square effect size (df)	*P* value
**Accurate response? (Yes)**		63/103 (61.2%)		
	Alexa	4/6 (66.7%)	4.558 (2)	.10
	Siri	26/36 (72.2%)	4.558 (2)	.10
	Cortana	33/61 (54.1%)	4.558 (2)	.10
**Evidence provided? (Yes)**		50/103 (48.5%)		
	Alexa	6/6 (100%)	11.054 (2)	.004
	Siri	21/36 (58.3%)	11.054 (2)	.004
	Cortana	23/61 (37.7%)	11.054 (2)	.004
**Misinformation provided? (Yes)**		18/103 (17.5%)		
	Alexa	0/6 (0%)	7.807 (2)	.02
	Siri	2/36 (5.6%)	7.807 (2)	.02
	Cortana	16/61 (26.2%)	7.807 (2)	.02

**Table 4:** Sentiment differences among the smart assistants.

Smart assistant	Content sentiment							*P* value
	Neutral (N=18)	Negative (N=7)	Ambiguous (N=11)	Positive (N=65)		Chi-square effect size (df)		
Alexa (N=6), n (%)	1 (16.7%)	0 (0%)	0 (0%)	5 (83.3%)		8.20 (6)		.22
Siri (N=35), n (%)	9 (25.7%)	0 (0%)	5 (14.3%)	21 (60%)		8.20 (6)		.22
Cortana (N=60), n (%)	8 (13.3%)	7 (11.7%)	6 (10%)	39 (65%)		8.20 (6)		.22

## Discussion

### Principal Results

This study sought to determine whether responses to queries vary among smart assistants in terms of accuracy, misinformation, and sentiment related to HPV vaccination. Our results indicate that smart assistants responded differently when asked about this topic. Specifically, smart assistants showed variations in the level of misinformation provided in responses, as well as the provision of evidence to support claims made in their responses. Smart assistants did not differ statistically in terms of the accuracy of their responses to queries involving HPV vaccination.

To our knowledge, this study is the first to report on smart assistants and their responses to HPV vaccine–related queries. Previous studies of HPV vaccination behaviors did not focus on smart assistants, and they indicated that antivaccination messages may influence one’s decisions to vaccinate themselves or their children [15]. However, given the growing number of persons and households adopting and utilizing smart assistants and the fact that smart assistants may be able to provide HPV vaccine information, it is necessary to examine whether responses delivered through smart assistants are disseminating these same antivaccination messages. This study is the first step in establishing a foundation for the types of vaccine sentiments that are present in content disseminated by smart assistants.

Importantly, our study revealed that, in general, smart assistants largely provide accurate and positive information regarding HPV vaccination (just under two-thirds of all smart assistant responses were accurate and positive), with no relevant differences across devices. We should reiterate here, however, that Google Assistant provided nonresponses to every query and, accordingly, was removed from the study. While accurate information on something as beneficial as HPV vaccination is necessary to mitigate HPV transmission and cancer incidence, just over one-third of responses contained *inaccurate* answers, suggesting that work is needed in this area to improve the provision of health information via smart assistants. On the other hand, misinformation was more of a concern across devices, as Cortana yielded the greatest number of responses containing misinformation (one-quarter of Cortana’s responses contained misinformation).

### Comparison With Prior Work

Previous studies of smart assistant responses have focused on whether the devices responded correctly or whether they understood the query [5,6]. Only a handful of studies examined smart assistant responses in the context of a health behavior [10]. This study further assessed smart assistant responses to determine whether they provide accurate evidence to specific health behavior questions. Evidence is critical in combatting misinformation, at least on social media platforms. For example, in an experimental study that exposed Facebook users to simulated misinformation and different correction mechanisms about the Zika virus, the authors found that correction can work when it provides supporting evidence or appropriate sources to accompany refutation (eg, Centers for Disease Control and Prevention) [36]. The authors reported that this finding was maintained even in those who were rated higher in conspiracy beliefs.

### Limitations

This study has limitations that should be considered when evaluating its results. The devices used their specific locations when responding to queries, which may have produced less generalizable responses. Some of the smart assistants employed in the study were previously used devices and may have been influenced by prior searches of the former user. To the best of our knowledge, no private mode exists in these smart assistants, which would prevent the queries and responses from influencing future searches. The sample size was limited for several reasons, including nonresponses and other unrelated responses to queries. Specifically, Google Assistant provided no response data, which may have influenced the results. Any responses that were entirely unrelated to the queries should be explored in future studies, since this may be an indication of a larger issue with smart assistants not comprehending the queries. Based on our knowledge about natural language processing in these smart assistants, it is suggested that syntax and dictation are some of the many influencers of smart assistant responses [37], and the queries posed to each device may not have been appropriately worded to elicit the most effective responses. For example, our use of a single investigator to ask the questions could be viewed as a limitation (ie, we do not know how the smart assistants would have responded to an investigator with a different gender, tone, or accent). The investigator only queried a single example of a smart assistant (ie, only one device was used to query Alexa), which could be a limitation, as smart assistants may respond differently depending on whether a stand-alone smart assistant or a smartphone-based smart assistant is used. The underlying search engine behind each smart assistant determines the responses to queries, which could have limited the information provided by smart assistants that are not connected to large search engines such as Google. The varying number of results provided in the smart assistant responses to queries could have impacted our ability to compare across the devices; however, since we found no relevant differences between the devices with regard to accuracy or sentiment, we do not believe that more results from one device impacted our findings or conclusions.

Despite these limitations, there are several strengths that provide further evidence of the study’s validity and importance. First, we utilized questions submitted to a Planned Parenthood chat service, which represent real-world issues on which consumers have previously sought additional information. Second, the questions used as queries were chosen because responses to these queries could be scored as either correct or incorrect, limiting “gray areas” in assessing accuracy of the smart assistant responses. Third, the queries were conducted by a single member of the research team to maintain consistency in language processing factors (eg, tone and syntax), and the search and coding processes were systematic to maintain consistency as well. Overall, this research furthers our understanding of how an emerging technology disseminates health information and whether such information can be classified as accurate and evidence-based. This study provides a basic framework for future evaluation of these smart assistants on which more advanced devices may be based.

Our findings paint a picture of smart assistants as newly emerged potential health promotion tools that are yet to be rigorously evaluated in this area. Smart assistants have only recently been introduced as potential health promotion tools (most notably from an executive perspective [38]) despite the growing proportion of households and individuals that use them [2,3]. The untapped potential of these devices for evidence-based information dissemination to consumers should be further explored. The potential of the devices may also be determined by their manufacturers who have, in some cases, provided platforms on which specialized topical information can be consolidated and further explored. For example, Alexa has an option for developers to create an Alexa Skill, which is essentially an application for Alexa that provides predefined information when queried specifically for that information. For example, an Alexa Skill that specifically searches in predefined evidence-based sources when queried for information on HPV vaccination could be developed. The downside to this potential approach for improving public health is that, as our results show, there is risk of disseminating misinformation through smart assistant responses, potentially reducing the positive impacts of a health promotion intervention. More needs to be done to better understand the susceptibility of these devices and their respective skills to outside influences.

### Conclusions

Our results suggest that not all smart assistants are created equal with regard to utility, at least when it comes to provided evidence and misinformation in their responses. These findings should spark further research into how the proprietors of smart assistants procure the information that is disseminated to consumers of their products. Specifically, we suggest that manufacturers of these and other smart assistants collaborate with researchers to further evaluate the accuracy of smart assistants as public health tools and determine together how to disseminate information and what fact-checking assessments should be used for such information. With the high rate of HPV transmission globally and in the United States, smart assistants and their manufacturers are well positioned to deliver evidence-based health information to consumers. However, such a practice necessitates strong communication between technology companies, who may not be as focused on the accuracy or source of their most heavily promoted content, and public health entities. The focus on collaboration to address the issues of information accuracy and misinformation is paramount if we are to adequately respond to the WHO’s list of urgent global health challenges for the new decade, namely stopping vaccine-preventable diseases.

## Appendix

Multimedia Appendix 1 Frequencies of responses and nonresponses by each smart assistant.

## Abbreviations

HPV: human papillomavirus
WHO: World Health Organization

## References

[1] Hoy MB (2018). Alexa, Siri, Cortana, and More: An Introduction to Voice Assistants. Med Ref Serv Q.

[2] (2017). Nearly half of Americans use digital voice assistants, mostly on their smartphones. Pew Research Center.

[3] Auxier B (2019). 5 things to know about Americans and their smart speakers. Pew Research Center.

[4] Fox S, Duggan M (2013). Health Online 2013. Pew Research Center.

[5] Berdasco A, López G, Diaz I, Quesada L, Guerrero LA (2019). User Experience Comparison of Intelligent Personal Assistants: Alexa, Google Assistant, Siri and Cortana. Proceedings.

[6] López G, Quesada L, Guerrero L (2018). Alexa vs. Siri vs. Cortana vs. Google Assistant: A Comparison of Speech-Based Natural User Interfaces.

[7] Michie S, Yardley L, West R, Patrick K, Greaves F (2017). Developing and Evaluating Digital Interventions to Promote Behavior Change in Health and Health Care: Recommendations Resulting From an International Workshop. J Med Internet Res.

[8] Triantafyllidis AK, Tsanas A (2019). Applications of Machine Learning in Real-Life Digital Health Interventions: Review of the Literature. J Med Internet Res.

[9] Zanaboni P, Ngangue P, Mbemba GI, Schopf TR, Bergmo TS, Gagnon M (2018). Methods to Evaluate the Effects of Internet-Based Digital Health Interventions for Citizens: Systematic Review of Reviews. J Med Internet Res.

[10] Boyd M, Wilson N (2018). Just ask Siri? A pilot study comparing smartphone digital assistants and laptop Google searches for smoking cessation advice. PLoS One.

[11] Senkomago V, Henley SJ, Thomas CC, Mix JM, Markowitz LE, Saraiya M (2019). Human Papillomavirus-Attributable Cancers - United States, 2012-2016. MMWR Morb Mortal Wkly Rep.

[12] Drolet M, Bénard É, Pérez N, Brisson M, HPV Vaccination Impact Study Group (2019). Population-level impact and herd effects following the introduction of human papillomavirus vaccination programmes: updated systematic review and meta-analysis. Lancet.

[13] Bradshaw AS, Treise D, Shelton SS, Cretul M, Raisa A, Bajalia A, Peek D (2020). Propagandizing anti-vaccination: Analysis of Vaccines Revealed documentary series. Vaccine.

[14] Del Vicario M, Bessi A, Zollo F, Petroni F, Scala A, Caldarelli G, Stanley HE, Quattrociocchi W (2016). The spreading of misinformation online. Proc Natl Acad Sci U S A.

[15] Dubé E, Vivion M, MacDonald NE (2015). Vaccine hesitancy, vaccine refusal and the anti-vaccine movement: influence, impact and implications. Expert Rev Vaccines.

[16] Elkin LE, Pullon SR, Stubbe MH (2020). 'Should I vaccinate my child?' comparing the displayed stances of vaccine information retrieved from Google, Facebook and YouTube. Vaccine.

[17] Hoffman BL, Felter EM, Chu K, Shensa A, Hermann C, Wolynn T, Williams D, Primack BA (2019). It's not all about autism: The emerging landscape of anti-vaccination sentiment on Facebook. Vaccine.

[18] Kata A (2012). Anti-vaccine activists, Web 2.0, and the postmodern paradigm--an overview of tactics and tropes used online by the anti-vaccination movement. Vaccine.

[19] Kata A (2010). A postmodern Pandora's box: anti-vaccination misinformation on the Internet. Vaccine.

[20] Southwell BG, Thorson EA, Sheble L (2017). The Persistence and Peril of Misinformation. Am. Sci.

[21] Cooper DL, Hernandez ND, Rollins L, Akintobi TH, McAllister C (2017). HPV vaccine awareness and the association of trust in cancer information from physicians among males. Vaccine.

[22] Fu LY, Zimet GD, Latkin CA, Joseph JG (2017). Associations of trust and healthcare provider advice with HPV vaccine acceptance among African American parents. Vaccine.

[23] Gidengil C, Chen C, Parker AM, Nowak S, Matthews L (2019). Beliefs around childhood vaccines in the United States: A systematic review. Vaccine.

[24] LaCour M, Davis T (2020). Vaccine skepticism reflects basic cognitive differences in mortality-related event frequency estimation. Vaccine.

[25] Thompson EL, Wheldon CW, Rosen BL, Maness SB, Kasting ML, Massey PM (2020). Awareness and knowledge of HPV and HPV vaccination among adults ages 27-45 years. Vaccine.

[26] (2020). Urgent health challenges for the next decade. World Health Organization.

[27] Paterson P, Meurice F, Stanberry LR, Glismann S, Rosenthal SL, Larson HJ (2016). Vaccine hesitancy and healthcare providers. Vaccine.

[28] Broniatowski DA, Jamison AM, Qi S, AlKulaib L, Chen T, Benton A, Quinn SC, Dredze M (2018). Weaponized Health Communication: Twitter Bots and Russian Trolls Amplify the Vaccine Debate. Am J Public Health.

[29] (2019). Ten health issues WHO will tackle this year. World Health Organization.

[30] Dibble KE, Maksut JL, Siembida EJ, Hutchison M, Bellizzi KM (2019). A Systematic Literature Review of HPV Vaccination Barriers Among Adolescent and Young Adult Males. J Adolesc Young Adult Oncol.

[31] Warner EL, Ding Q, Pappas L, Bodson J, Fowler B, Mooney R, Kirchhoff AC, Kepka D (2017). Health Care Providers' Knowledge of HPV Vaccination, Barriers, and Strategies in a State With Low HPV Vaccine Receipt: Mixed-Methods Study. JMIR Cancer.

[32] Olson C, Kemery K (2019). 2019 Voice report: Consumer adoption of voice technology and digital assistants. Microsoft.

[33] Petrescu P, Ghita M, Loiz D (2014). Google Organic CTR Study. Advanced Web Ranking.

[34] Lincoln J (2020). SEO and Intent 2020: New Study Reveals True State Of Search. Ignite Visibility.

[35] Landis JR, Koch GG (1977). The Measurement of Observer Agreement for Categorical Data. Biometrics.

[36] Bode L, Vraga EK (2018). See Something, Say Something: Correction of Global Health Misinformation on Social Media. Health Commun.

[37] Goksel Canbek N, Mutlu ME (2016). On the track of Artificial Intelligence: Learning with Intelligent Personal Assistants. HumanSciences.

[38] Cimino J, Stefanacci R, Pakizegee M (2020). Alexa, Can You Transform Healthcare?. Managed Healthcare Executive.

