# Supplementary figures and images for: Evaluating Smart Assistant Responses for Accuracy and Misinformation Regarding Human Papillomavirus Vaccination: Content Analysis Study
Source: J Med Internet Res. 2020 Aug 3;22(8):e19018. doi: 10.2196/19018 (PMC7432152; doi:10.2196/19018)

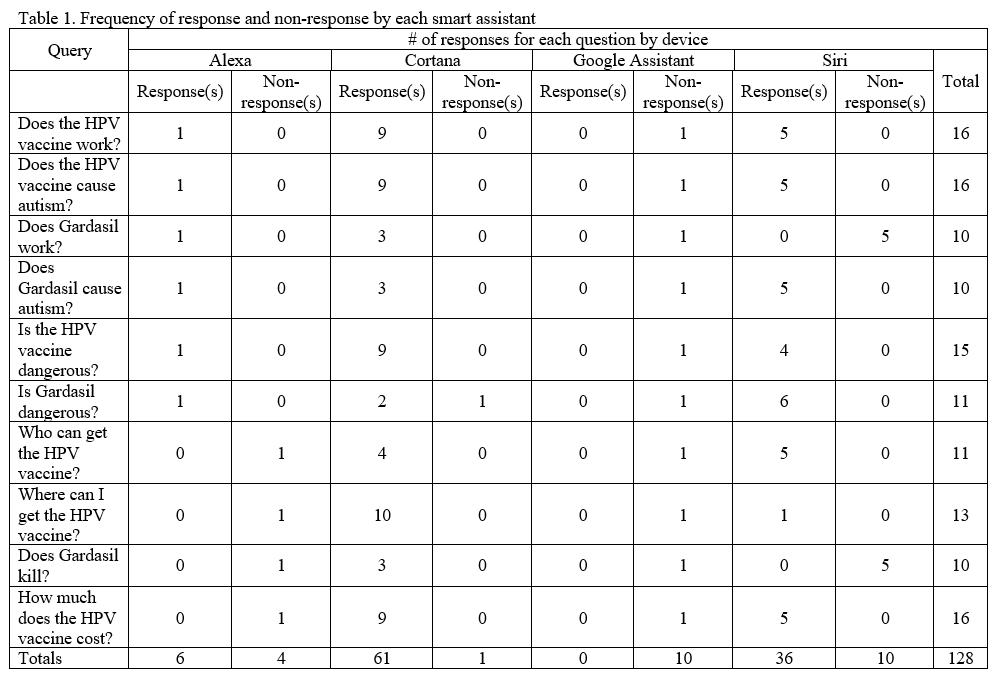

Supplement: Multimedia Appendix 1 [file jmir_v22i8e19018_app1.PNG]
